# Validation of risk prediction models applied to longitudinal electronic health record data for the prediction of major cardiovascular events in the presence of data shifts

**DOI:** 10.1093/ehjdh/ztac061

**Published:** 2022-10-21

**Authors:** Yikuan Li, Gholamreza Salimi-Khorshidi, Shishir Rao, Dexter Canoy, Abdelaali Hassaine, Thomas Lukasiewicz, Kazem Rahimi, Mohammad Mamouei

**Affiliations:** Deep Medicine, Oxford Martin School, University of Oxford, Hayes House, 75 George Street, Oxford OX1 2BQ, UK; Nuffield Department of Women’s and Reproductive Health, Medical Science Division, University of Oxford, Oxford, UK; Deep Medicine, Oxford Martin School, University of Oxford, Hayes House, 75 George Street, Oxford OX1 2BQ, UK; Nuffield Department of Women’s and Reproductive Health, Medical Science Division, University of Oxford, Oxford, UK; Deep Medicine, Oxford Martin School, University of Oxford, Hayes House, 75 George Street, Oxford OX1 2BQ, UK; Nuffield Department of Women’s and Reproductive Health, Medical Science Division, University of Oxford, Oxford, UK; Deep Medicine, Oxford Martin School, University of Oxford, Hayes House, 75 George Street, Oxford OX1 2BQ, UK; Nuffield Department of Women’s and Reproductive Health, Medical Science Division, University of Oxford, Oxford, UK; NIHR Oxford Biomedical Research Centre, Oxford University Hospitals NHS Foundation Trust, Oxford, UK; Deep Medicine, Oxford Martin School, University of Oxford, Hayes House, 75 George Street, Oxford OX1 2BQ, UK; Nuffield Department of Women’s and Reproductive Health, Medical Science Division, University of Oxford, Oxford, UK; Department of Computer Science, University of Oxford, Oxford, UK; Deep Medicine, Oxford Martin School, University of Oxford, Hayes House, 75 George Street, Oxford OX1 2BQ, UK; Nuffield Department of Women’s and Reproductive Health, Medical Science Division, University of Oxford, Oxford, UK; NIHR Oxford Biomedical Research Centre, Oxford University Hospitals NHS Foundation Trust, Oxford, UK; Deep Medicine, Oxford Martin School, University of Oxford, Hayes House, 75 George Street, Oxford OX1 2BQ, UK; Nuffield Department of Women’s and Reproductive Health, Medical Science Division, University of Oxford, Oxford, UK

**Keywords:** Cardiovascular disease risk, Heart Failure, Stroke, Coronary heart disease, Predictive modelling, Data shifts

## Abstract

**Aims:**

Deep learning has dominated predictive modelling across different fields, but in medicine it has been met with mixed reception. In clinical practice, simple, statistical models and risk scores continue to inform cardiovascular disease risk predictions. This is due in part to the knowledge gap about how deep learning models perform in practice when they are subject to dynamic data shifts; a key criterion that common internal validation procedures do not address. We evaluated the performance of a novel deep learning model, BEHRT, under data shifts and compared it with several ML-based and established risk models.

**Methods and results:**

Using linked electronic health records of 1.1 million patients across England aged at least 35 years between 1985 and 2015, we replicated three established statistical models for predicting 5-year risk of incident heart failure, stroke, and coronary heart disease. The results were compared with a widely accepted machine learning model (random forests), and a novel deep learning model (BEHRT). In addition to internal validation, we investigated how data shifts affect model discrimination and calibration. To this end, we tested the models on cohorts from (i) distinct geographical regions; (ii) different periods. Using internal validation, the deep learning models substantially outperformed the best statistical models by 6%, 8%, and 11% in heart failure, stroke, and coronary heart disease, respectively, in terms of the area under the receiver operating characteristic curve.

**Conclusion:**

The performance of all models declined as a result of data shifts; despite this, the deep learning models maintained the best performance in all risk prediction tasks. Updating the model with the latest information can improve discrimination but if the prior distribution changes, the model may remain miscalibrated.

## Graphical Abstract

**Graphical Abstract:**
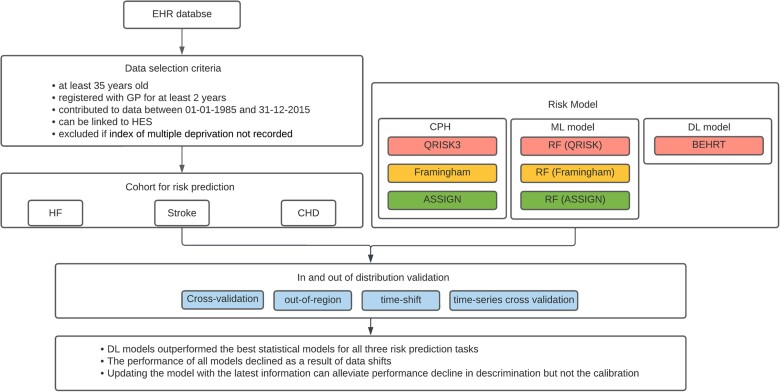
Design and main results of the model evaluation in the presence of data shift. EHR, electronic health records; HES, hospital episode statistics; HF, heart failure; CHD, coronary heart disease; CPH, COX proportional hazard; ML, machine learning; DL, deep learning; RF, random forest.

Translational perspective

Cardiovascular disease (CVD) risk models have a long tradition in clinical care. Despite rapid advances in modelling over the past decade and the increasing availability of longitudinal electronic health records, CVD risk models proposed in 1990s and 2000s are still routinely used across the world with minor changes. In line with the Transparent reporting of a multivariable prediction model for individual prognosis or diagnosis (TRIPOD) statement and using representative data from 3.05 million individuals in the UK, we performed a rigorous comparison of novel deep learning and machine learning models with conventional CVD risk models. The findings highlight the merits and shortcomings of these models in terms of predictive performance.

## Introduction

Risk prediction models are important tools to guide decision-making in routine health care. They can help clinicians to identify at-risk patients and initiate preventive measures. Today, most prediction models in use, rely on (simple) statistical techniques with expert selected predictors. For example, QRISK3,^1^ Framingham,^2^ and ASSIGN^3^ are commonly used risk models for the prediction of cardiovascular events. These models largely assume a linear relationship between covariates and outcomes, and this assumption is shown to limit the predictive power and accuracy in some applications.^4–6^

Over the past decade, deep learning (DL) models have gained growing popularity due to their ability to learn high-level features that capture the complex interactions of input variables. DL models have delivered state-of-the-art predictive performance across different fields without the need for expert-guided feature engineering.^4,7^ Indeed, several previous studies demonstrated that simpler machine learning (ML) models could outperform statistical models and suggested their advantages in improving healthcare.^8,9^ However, several factors have hampered the adoption of more complex DL models for CVD risk assessment in healthcare.

The explainability of deep over-parametrized models remains an open question and an active area of research. However, a more fundamental question has cast a shadow over the usability of these models in clinical practice. Some have argued that beyond internal validations that are prone to being optimistic and are not representative of the practical use cases of risk models, evidence of achieving higher performance using DL models is lacking. Other studies have shown that simple statistical models can perform better or as well as ML models.^10,11^ These contradictory findings are partly because in the absence of benchmarking datasets, the data used in different studies—in terms of size and quality—may not always merit complex modelling approaches. More importantly, model evaluation methods and metrics vary across different studies. Lastly, DL models encompass a wide range of architectures and depending on the data the benefits of different architectures can vary significantly.^7,12^

The objective of this study is to compare the performance of a novel DL model, trained on large-scale, representative electronic health records (EHRs), with three widely used Cox proportional hazards (CPH) models for prediction of risk of cardiovascular disease (QRISK, Framingham, and ASSIGN). A more conventional ML model (random forest) was also included in the analysis as an additional comparator. As case studies, we investigated the prediction of major cardiovascular events [heart failure (HF), stroke, and coronary heart disease (CHD)] in the general population. Considering out-of-distribution dataset shift is an inherent problem that applies to all modelling approaches, to better assess these models’ generalization power and robustness, in addition to internal validation, we devised a number of ‘external’ validations procedures. Specifically, we investigated model performance under data shifts on external cohorts (1) from different geographies, and (2) from different time periods.

## Methods

### Study design and participants

**Figure 1:**
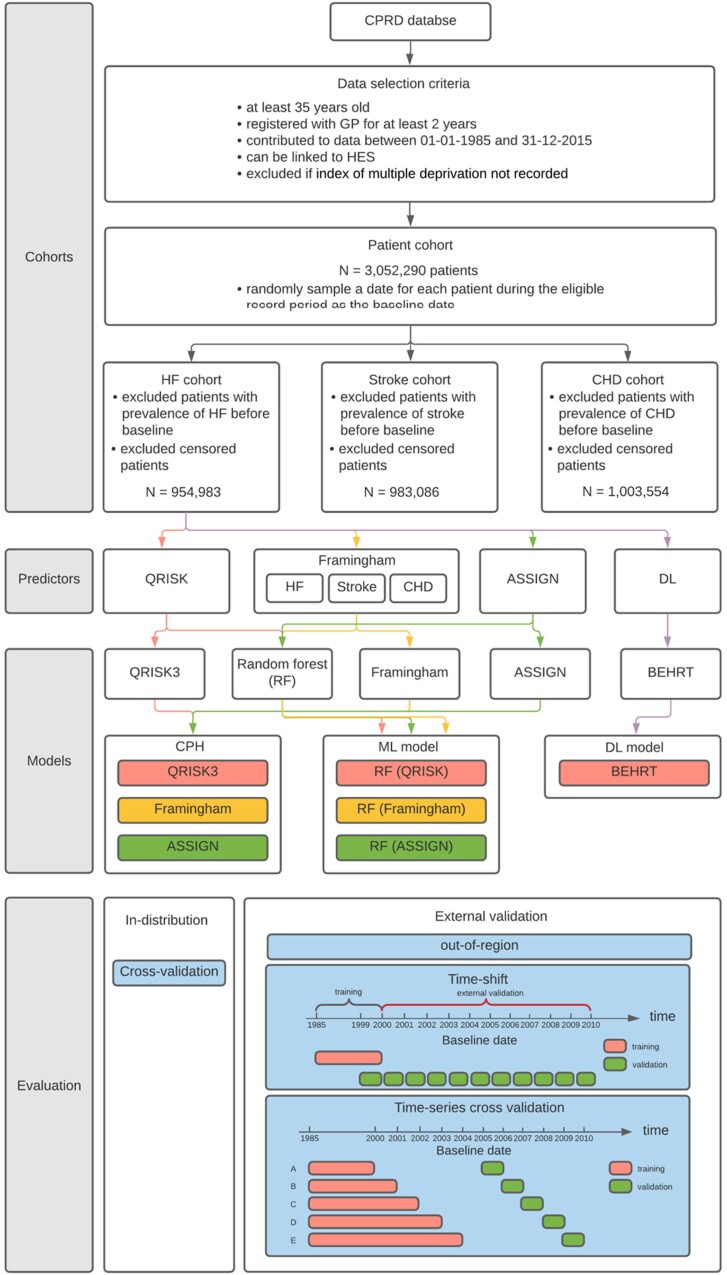
Flow diagram of the cohort selection, modelling, and evaluation. Heart failure is used for demonstration of the seven models for comparison, but the Stroke and coronary heart disease risk prediction follow the same protocol. The colour in predictor and modelling section represents a path that flows from the cohort to a specific set of predictors and how the predictors are used by the models.

The Clinical Practice Research Datalink (CPRD)^13^ provides de-identified patient data collected from general practices (GP) across the UK. The primary care data can be linked to other health-related data such as the Hospital Episode Statistics (HES) Admitted Patient Care data and the death registration data from the Office for National Statistics.^14^ It is broadly representative of the UK population, and it is one of the most comprehensive EHR datasets. To create our study dataset, we selected a subset of patients, both men and women, aged at least 35 years, registered with GP for at least two years, contributed to data between 01 January 1985 and 32 December 2015 and were linked to HES. Patients were excluded if they had no index of multiple deprivation (IMD), which is an area-based socioeconomic status indicator, with increasing level of deprivation with higher scores (*Figure 1*). We determined a baseline date for each patient by randomly selecting a date during the eligible record period. This approach can better capture the practice variability with a better spread of calendar time and age.^11,15^

This work mainly investigated the 5-year risk prediction of the incidence of three major cardiovascular events, namely HF, stroke, and CHD. To identify cases for each risk prediction task, for example HF, we filtered out all patients with prevalent HF. A patient was identified as HF (+) if diagnosed with HF from either GP and HES records or death registration within a 5-year interval after the baseline date. Moreover, a patient was identified as HF (−) if one had records for at least 5 years after the baseline and there was no HF identified during the 5-year interval. The rest of patients were considered as lost to follow-up before developing HF by year 5 with uncertain outcome (i.e. censored patients), thus, were excluded from the cohort. We followed the identical protocol to prepare the dataset for stroke or CHD risk prediction. The diagnosis codes for HF, stroke, and CHD were adapted from the CALIBER code repository.^16^ In brief, HF was defined as a composite condition of congestive HF, left ventricular failure, cor pulmonale, cardiomyopathy, hypertensive heart disease with (congestive) HF, cardiac failure, and HF; stroke was defined as a composite condition of ischaemic stroke, transient ischaemic attack, and stroke not otherwise specified; and CHD was defined as a composite condition of atherosclerotic heart disease, coronary or ischaemic heart disease, aborted myocardial infarction, or coronary artery disease.

### Predictor variables

We used six sets of predictors (input variables to the predictive models) for risk prediction. Two of them were for QRISK and ASSIGN,^1,3^ respectively, and they were used for all three risk prediction tasks. As suggested by the previous research,^2,17–19^ Framingham model used three different sets of predictors for HF,^17,18^ stroke,^19^ and CHD^2^ risk prediction, respectively. Another set of predictors was used for training the DL models. *Table 1* shows the complete list of predictors except for DL. Missing values were imputed using Multivariable imputation with chained equations^20^ for five times. More details about data missingness, predictor extraction, and data imputation can be found in the Supplementary Material online.

**Table 1:** Summary of predictors

Predictor	QRISK	Framingham-HF	Framingham-Stroke	Framingham-CHD	ASSIGN
Age	*	*	*	*	*
Ethnicity	*				
Indices of multiple deprivations	*				*
Systolic blood pressure	*	*	*	*	*
SD of systolic blood pressure	*				
Body mass index	*				
Total cholesterol/HDL ratio	*				*
Smoking status	*		*	*	*
Family history of CHD	*				*
Diabetes	*	*	*		*
Treated hypertension	*		*	*	
Rheumatoid arthritis	*				*
Atrial fibrillation	*		*		
CKD (stage 3, 4, 5)	*				
Migraine	*				
Corticosteroid use	*				
Systemic lupus erythematosus	*				
Atypical antipsychotic drugs	*				
Severe mental illness	*				
HIV or AIDS	*				
Erectile dysfunction	*				
Sex		*		*	*
CHD		*	*		
Left ventricular hypertrophy		*			
Valve disease		*			
Heart rate		*			
Total cholesterol				*	
HDL				*	

CKD, chronic kidney disease; * represents predictors selected by each model.

We did not explicitly select predictors for the DL model. The model was trained end-to-end on raw (or minimally processed) EHR for the risk prediction tasks without any imputation. We incorporated all diagnoses, medications, lab tests, and procedures available before baseline with 3858, 390, 1439, and 679 distinct medical codes in each data category respectively (see Supplementary Material online).

### Derivation of the models

This study considered seven models for each of the risk prediction tasks (*Figure 1*), including a DL model^7^ (i.e. BEHRT), three CPH models (QRISK3,^1^ Framingham,^2,17–19^ and ASSIGN^3^) from ‘Lifelines’,^21^ and three random forest models^22^ from ‘scikit-learn’^23^ that relied on predictors selected by QRISK3, Framingham, and ASSIGN, respectively. We referred to these three random forest models as RF (QRISK), RF (Framingham), and RF (ASSIGN) based on the selection of their predictors. We used RF instead of the survival RF in our work because the current software packages of survival RF cannot handle large data very well to our best knowledge. The Framingham family of CVD models has a different set of predictors for HF, Stroke, and CHD. This distinction is reflected in the naming of the models in the present study. On the contrary, QRISK and ASSIGN are used for general CVD prediction. In the present study we used the same set of predictors for the prediction of all three diseases. BEHRT is a recently proposed sequential DL model that uses Transformer^24^ and a self-attention mechanism^24^ to extract the temporal patterns within a sequence. It learns the health trajectory of patients by modelling the EHR in chronological order. This distinguishes BEHRT from the other models in the present study. Statistical models measure risk on the basis of the absence or presence of a disease with no or limited interactions. BEHRT in addition to capturing complex interactions, incorporates in the prediction of risk the order in which medical events such as diagnoses and treatments were observed, the age at which an individual has received a diagnosis and other medical events in a patient’s medical records (see Supplementary Material online).

For ML and DL models, we performed hyperparameter tuning by randomly searching a given parameter space for 30 iterations. For brevity, we only reported the model with the best results and the selected values for the parameters (see Supplementary Material online). Additionally, for clarification, all models (including QRISK, Framingham, and ASSIGN) were implemented, trained, and validated using the selected cohort. Also, no class imbalance correction was applied for model training.^25^

### Model evaluation and statistical analysis

We employed a range of model evaluation procedures to compare the performance of models beyond the narrowly defined internal validation procedures (*Figure 1*). In addition to a standard five-fold cross-validation (or ‘internal validation’), we investigated three ‘external validation’ approaches. First, for the out of region validation, we followed the recommendations^9^ and chose different regions (i.e. strategic health authorities) for forming the training and validation datasets (i.e. regions in the validation dataset, were not part of the training data). More specifically, for validation sets we selected three regions with different socioeconomic statuses^9^ (see Supplementary Material online) from north England that account for 23% of the total population. The remaining regions were used for training. We argue that geographically different patients with different statistical characteristics are similar to using an external dataset with a certain level of data shift.^9^

Second, for the temporal (i.e. time-shift) validation, we expect that changes in demographics, care policy and practice over years to introduce data shifts.^26^ Therefore, we trained and validated models on cohorts from different years. More specifically, we randomly chose a baseline for each individual. All those with baseline years of 2000 and earlier were used for training models and those with baseline year between 2000 and 2010 were used for validation (*Figure 1*). Additionally, by evaluating the model performance on patients from baseline year 1999–2000, we created an internal reference to compare with the model performance under temporal changes. Thus, this evaluation can provide a clear trend on how model performance changes for datasets without (i.e. internal reference) or with data shifts in terms of temporal variability. The baseline characteristics can be found in the Supplementary Material online.

In routine clinical settings, a risk prediction model can only be trained with the data in the past to predict an event in the future. Assume we want to use a risk model in year 2005; this means we need a model that has been trained and validated with data before 2005. To assess the models’ generalization power in settings similar to their typical use case, and over a period of time, our third external validation performed ‘time-series cross-validation’.^27^ In this approach, we split the data into three time-windows, denoted as *w*_*train*_, *w*_*label*_, and *w*_*val*_. For example, we trained a model using patients with baseline years before 2000 (*w*_*train*_), left a 5-year gap (2000–2005) for labelling (*w*_*label*_), and validated (*w*_*val*_) on patients with baseline one year after the labelling window (2005–2006). The time-series cross-validation repeated the abovementioned process by moving *w*_*train*_ and *w*_*eval*_ from 2000 to 2004, and 2006 to 2010, respectively, with a one-year interval. That is, a total of five models trained on five different training sets (five time windows) and evaluated on five separate validation sets.

While our outcome was binary, instead of predicting positive or negative, we predicted the probability of a patient that belongs to positive or negative. This allows us to measure the discrimination of models using area under the receiver operating characteristic curve (AUROC) and average precision (AP). The model calibration was assessed with the calibration curves.^28^ The DL and ML model can predict the estimated risk naturally within the binary classification framework. However, for the CPH survival models, we combined the regression coefficients as weight with the baseline survival function. It allowed us to estimate the risk for each year of follow-up, and we only focused on the 5-year risk estimation.^1^ This allows us to calculate the AUROC, AP, and calibration curves that are directly comparable with the binary classification models. Additionally, the precision related metrics (e.g. AP) were not considered for evaluation in the presence of data shifts as they are sensitive to the changes of event rate. Naturally, the event rate can change across time for all outcomes; therefore, these metrics are not comparable in this case.

To provide additional information about how the time shift influences the risk prediction, we analyzed the incidence rate of HF, stroke, and CHD across different years on the selected cohort. Furthermore, we used population stability index (SPI) to identify the covariates distribution shift^29^ between the training and validation dataset.

## Results

### Study population

Overall, 4 528 376 patients contributed to CPRD between 01 January 1985 and 31 December 2015 can be linked to HES and had IMD recorded. Among those, 3 902 516 patients were registered with the general practices for at least two years. With the inclusion of patient aged at least 35, we identified a cohort with 3 052 290 patients. Additionally, we filtered out patients with prior prevalent HF, stroke, and CHD before the baseline date for the prediction of incident HF, incident stroke, and incident CHD, respectively. This led to 3 009 580, 2 972 045, and 2 915 645 patients accordingly for the risk prediction tasks. With the exclusion of censored patients, we identified 954 983, 983 086, and 1 003 554 patients for HF, stroke, and CHD datasets, respectively, which were substantially smaller than the overall cohort. Due to the large overlap of cohorts across three risk prediction tasks, we summarized the baseline characteristics of the selected cohorts after data imputation together to simplify the presentation, and they are shown in *Table 2*.

**Table 2:** Baseline characteristics of risk prediction cohorts

Predictor	Cohort
No. of patients	1 096 275
No. of patients (% of positive cases) for HF cohort	954 983 (3.3)
No. of patients (% of positive cases) for Stroke cohort	983 086 (6.7)
No. of patients (% of positive cases) for CHD cohort	1 003 554 (10.3)
Sex, *n* (%)	
Male	534 657 (48.7)
Female	561 618 (51.2)
Age, mean (SD)	58.0 (15.3)
IMD score, mean (SD)	2.7 (1.4)
Family history of CHD, *n* (%)	341 042 (31.1)
BMI, mean (SD)	27.6 (3.2)
Strategic health authority (region), *n* (%)	
North East	27 195 (2.5)
North West	175 416 (16.0)
Yorkshire and the Humber	54 655 (5.0)
East Midlands	38 242 (3.5)
West Midlands	137 824 (12.6)
East of England	134 314 (12.3)
South West	130 170 (11.9)
South Central	136 189 (12.4)
London	126 175 (11.5)
South East Coast	136 047 (12.4)
Ethnicity, *n* (%)	
Unknown	683 961 (62.4)
White	399 533 (36.4)
Other Asian	1016 (0.1)
Pakistani	1237 (0.1)
Indian	3070 (0.3)
Other	3162 (0.3)
Caribbean	1659 (0.2)
Mixed	790 (0.1)
Bangladeshi	338 (0.0)
Chinese	656 (0.1)
Black African	853 (0.1)
Smoking status, *n* (%)	
Not recorded	776 927 (70.9)
Non-smoker	154 991 (14.1)
Ex-smoker	106 512 (9.7)
Light smoker (<10 cigarettes/day)	15 576 (1.4)
Moderate smoker (10–20 cigarettes/day)	23 269 (2.1)
Heavy smoker (>20 cigarettes/day)	19 000 (1.7)
Clinical values	
Systolic blood pressure, mean (SD)	135.3 (13.0)
Cholesterol/HDL	3.99 (0.67)
Comorbidity, *n* (%)	
Diabetes	38 972 (3.6)
Rheumatoid arthritis	7 507 (0.7)
Atrial fibrillation	34 137 (3.1)
Chronic kidney disease	6727 (0.6)
Migraine	34 789 (3.2)
Severe mental illness	6113 (0.5)
Systemic lupus erythematosus	947 (0.1)
HIV or AIDS	1755 (0.2)
Erectile dysfunction	27 885 (2.5)
Prescribed medication, *n* (%)	
Treated hypertension	233 056 (21.3)
Antipsychotic	5778 (0.5)
Corticosteroid	44 388 (4.0)

IMD, index of multiple deprivation; SD, standard deviation.

### Internal validation

**Figure 2:**
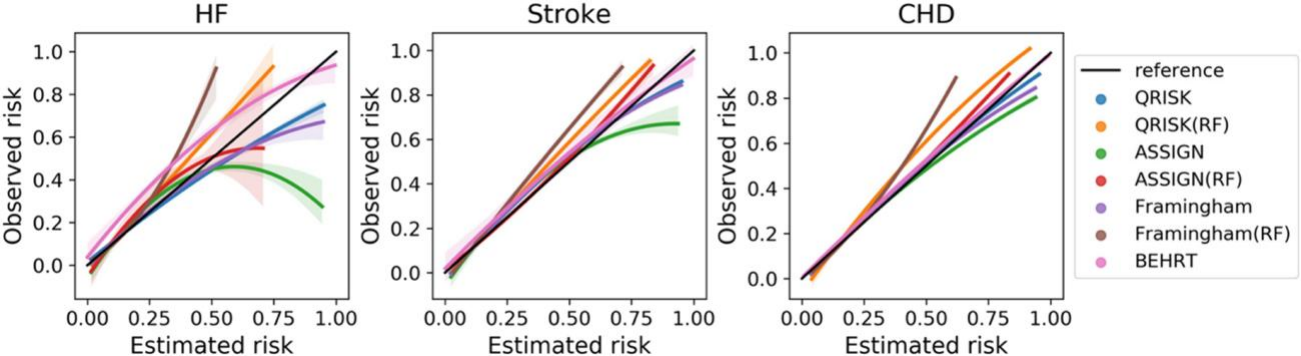
Mean and 95% confidence interval of calibration curve from five-fold internal cross validation.

*Table 3* shows the model performance of the statistical, ML, and DL models for HF, stroke, and CHD risk prediction. The BEHRT models substantially outperformed the other ML and statistical models for all risk prediction tasks, achieving AUROC 0.954 (95% confidence intervals: 0.950–0.958), 0.957 (0.955–0.959), and 0.951 (0.949–0.053); AP 0.801 (0.781–0.821), 0.876 (0.865–0.887), and 0.897 (0.884–0.910) for HF, stroke, and CHD risk prediction, respectively. Furthermore, ML models (i.e. random forest) showed similar or slightly better performance than the statistical models with the same predictors. *Figure 2* also shows that BEHRT models in general have better calibration performance than the other models, especially for the high-risk predictions, in all tasks.

**Table 3:** Mean and 95% confidence interval of AUROC and AP from five-fold internal cross-validation

Models	AUROC (95% confidence interval)		
	HF	Stroke	CHD
BEHRT	0.954 (±0.004)	0.957 (±0.002)	0.951 (0.002)
QRISK3	0.895 (±0.002)	0.874 (±0.005)	0.838 (±0.005)
RF (QRISK)	0.897 (±0.005)	0.877 (±0.005)	0.850 (±0.003)
ASSIGN	0.885 (±0.002)	0.85±8 (±0.002)	0.829 (±0.001)
RF (ASSIGN)	0.884 (±0.005)	0.859 (±0.003)	0.833 (±0.002)
Framingham	0.883 (±0.004)	0.869 (±0.005)	0.831 (±0.005)
RF (Framingham)	0.884 (±0.002)	0.868 (±0.004)	0.836 (±0.003)
AP (95% confidence interval)			
BEHRT	0.801 (±0.020)	0.876 (±0.011)	0.897 (±0.013)
QRISK3	0.270 (±0.002)	0.435 (±0.007)	0.463 (±0.002)
RF (QRISK)	0.313 (±0.031)	0.471 (±0.008)	0.487 (±0.006)
ASSIGN	0.230 (±0.006)	0.360 (±0.004)	0.386 (±0.005)
RF (ASSIGN)	0.278 (±0.007)	0.368 (±0.008)	0.398 (±0.008)
Framingham	0.270 (±0.002)	0.451 (±0.007)	0.431 (±0.007)
RF (Framingham)	0.302 (±0.016)	0.452 (±0.007)	0.442 (±0.006)

### External validation in regions

The AUROC values validated on the external cohort with patients from the non-random selected regions are reported in *Table 4*. All models showed a slight decline in AUROC compared to the internal validation, and BEHRT models generally showed worse degradations than the other ML and statistical models. On average, the decrease in AUROC was less than 1.5% for both ML and statistical models and around 3% for the BEHRT models. Yet, the BEHRT models still achieved the best performance for all risk prediction tasks.

**Table 4:** Externally validated model performance on a cohort with patients from the non-random selected regions

Models	AUROC (absolute decline compared to the internal performance)		
	HF	Stroke	CHD
BEHRT	0.909 (−0.044)	0.932 (−0.025)	0.929 (−0.022)
QRISK3	0.883 (−0.012)	0.865 (−0.009)	0.830 (−0.008)
RF (QRISK)	0.883 (−0.014)	0.866 (−0.011)	0.840 (−0.010)
ASSIGN	0.873 (−0.012)	0.852 (−0.003)	0.823 (−0.006)
RF (ASSIGN)	0.874 (−0.010)	0.853 (−0.006)	0.827 (−0.006)
Framingham	0.871 (−0.012)	0.862 (−0.007)	0.821 (−0.010)
RF (Framingham)	0.873 (−0.011)	0.855 (−0.013)	0.826 (−0.010)

### Impact of temporal data shift

**Figure 3:**
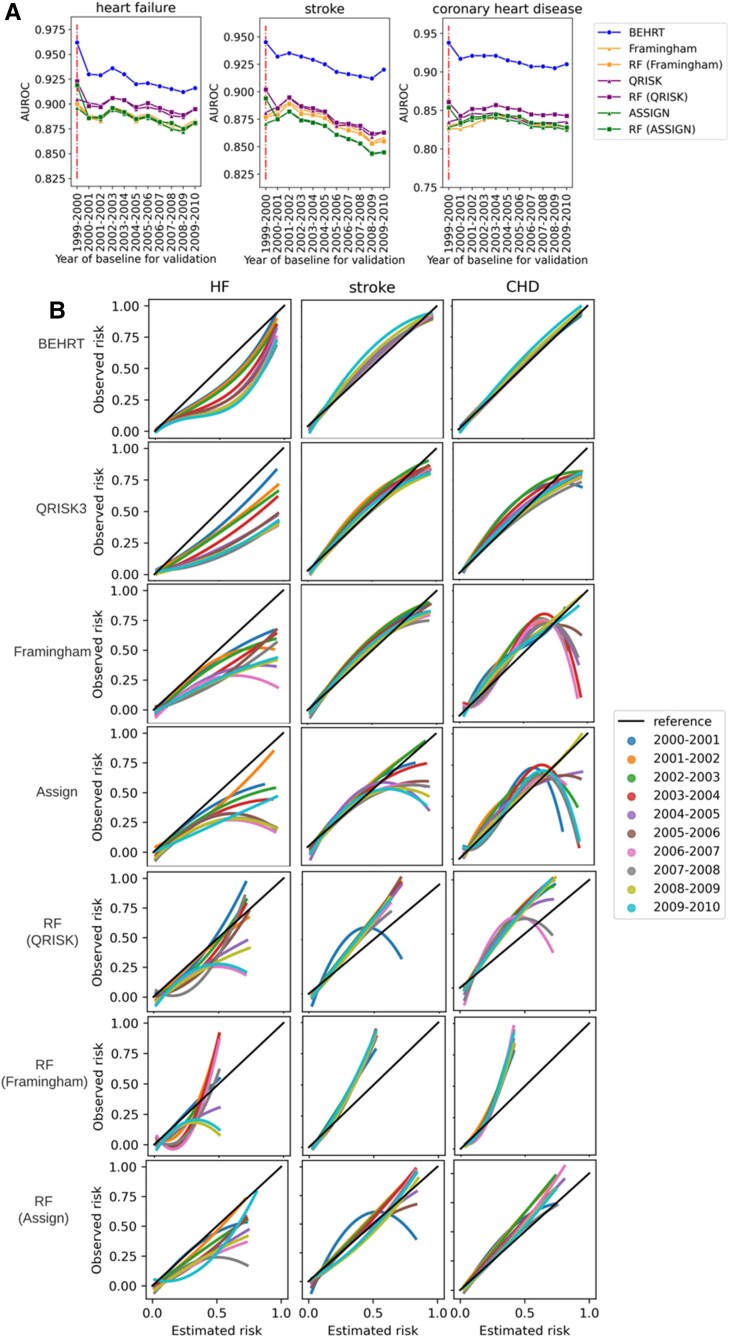
Model performance validated under temporal data shift. (*A*) Validation of discrimination. (*B*) Validation of calibration. All models were trained on patients who had baseline before 2000 and validated on patients who had baseline between 2000 and 2010 with 1-year interval. The internally validated AUROC (1999-2000) is provided for comparison in (*A*); and the diagonal line in (*B*) is the reference calibration curve. Colours in (*A*) represent the models for validation and the colours in (*B*) represent the year of baseline for validation. (*A*) Shows all models suffer performance decline under temporal data shifts. This is represented as AUROC values decrease as the gap between the reference (i.e. 1999–2000) and the baseline of the validated cohort increases. *S*imilarly, as the gap increases, the notable deviations of the calibration curve under temporal shifts for some models in (*B*) show they are more prone to the detrimental effects of data shifts.

*Figure 3A* shows the AUROC of all models when predicting the outcome for patients with external baseline. In general, all models suffered a performance decline under temporal data shift, and the AUROC values from all models decreased as the gap between the reference (i.e. 1999–2000) and the baseline of the validated cohort increased. Noticeably, HF and stroke had worse decline compared to CHD. Furthermore, there was no substantial difference between statistical models (i.e. Framingham, QRISK, and ASSIGN) and the ML models in terms of the external performance. The QRISK model in general has the best performance among the statistical models. Additionally, we see the BEHRT models and the ML models have a more considerable decline between internal (i.e. 1999–2000) and external (i.e. 2000–2010) performance. Additionally, *Figure 3B* shows the calibration curve of models under temporal data shift. The result shows that the temporal data shift can negatively affect the calibration of models. These negative effects are more pronounced in the calibration of the HF risk prediction models relative to stroke and CHD. However, BEHRT models still achieved the best calibration among all models for all three risk prediction tasks. This was followed by QRISK. The two models substantially outperformed the others, especially for CHD risk prediction. An important difference between the BEHRT and QRISK models pertains their performance in the high-risk region of the calibration curve. The QRISK models have relatively poor calibration in this region, especially under temporal data shift.

**Figure 4:**
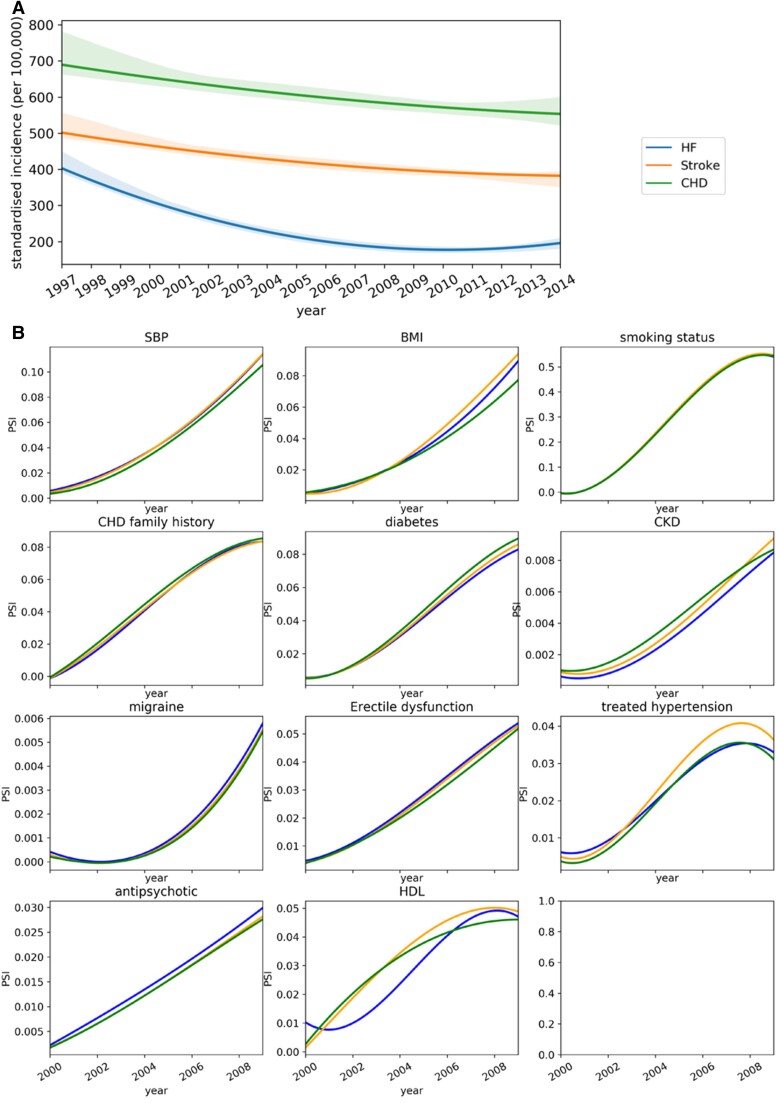
Analysis of data shift in the covariates and the outcome. (*A*) Temporal trends of incidence rate presented with fitted local polynomial regression lines with 95% confidence interval. The presented period covers the 95% patients’ baseline in the selected cohort. (*B*) Population stability index of predictors between training and temporal validation set (predictors with population stability index > 0.001 are presented). The colours in both (*A*) and (*B*) represent the diseases.

Data shifts are commonly classified into three categories: covariate shift (i.e. distribution shift in the covariates), prior probability shift (i.e. distribution shift in the outcome), and concept shift (i.e. the change of relation between covariates and the outcome).^30^ We specifically investigated the prior probability shift and covariate shift as shown in *Figure 4*. *Figure 4A* shows the incidence rate of HF substantially changes across different year (prior probability shifts), but it is not the case for CHD and stroke. Therefore, the prior probability shifts can be a potential reason that causes the calibration shift in HF as shown in *Figure 3B* (i.e. HF had severe calibration shift compared to stroke and CHD). *Figure 4B* shows the covariate shifts in general are not substantial (i.e. PSI < 0.1) for all three diseases, except systolic blood pressure and smoking status. Considering the performance decline as shown in *Figure 3A*, the concept shift can potentially play an important role in temporal data shift, and it suggests that the relation between covariates and the outcome can change over time.

### Time-series cross-validation

**Figure 5:**
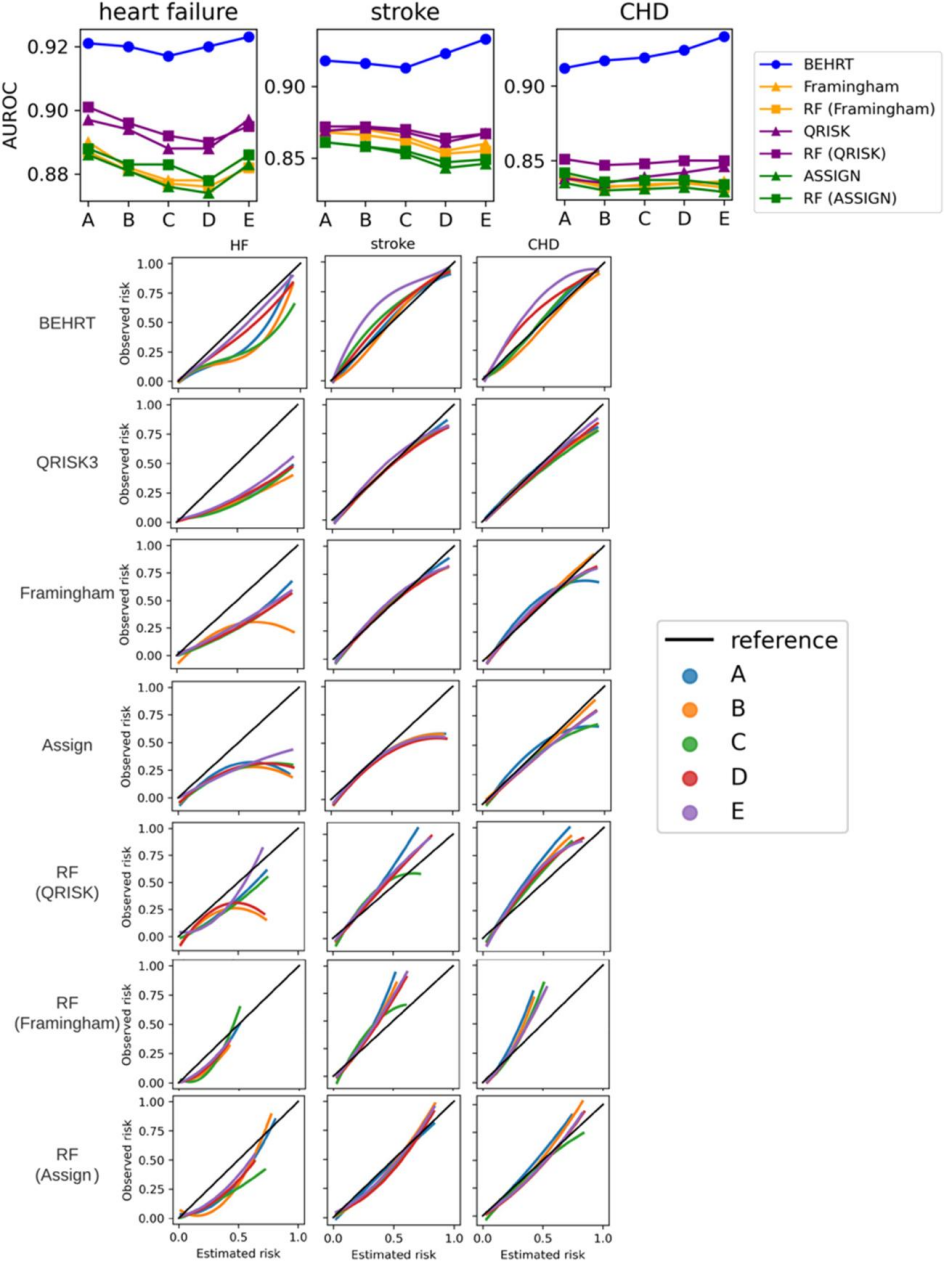
Area under the receiver operating characteristic curve and calibration curve using time series cross-validation. (*A*–*E*) represent experiments with *w*_*train*_ from 2000 to 2004 and *w*_*eval*_ from 2006 to 2010, respectively (*Figure 1*).

*Figure 5* shows the AUROC is relatively stable with less than 2% changes for all risk prediction tasks across different time, therefore, suggesting that all models benefit from using updated information to prevent performance decline in AUROC. However, this has negligible impact on the calibration curve. The calibration curve of the models for CHD risk prediction improves slightly more than HF. This is partially because using the updated information can mitigate concept shift (i.e. relation between the covariates and the outcome), but not the prior probability shift (i.e. distribution shift in the outcome). Therefore, the result suggests that updating model only improves the calibration when the prior probability shift is not severe.

## Discussion

Previous studies have applied ML methods to predict major cardiovascular events and reported comparisons with the established statistical models.^11,31,32^ However, the findings were largely based on internal validation and the generalizability of different models on the external cohorts were not addressed.^8,33,34^ Moreover, despite the importance of DL models and continued advances in this subfield of artificial intelligence, evidence of their relative benefits was scarce or limited to simple multi-layer perceptron networks.^11,34,35^ There is a gap in knowledge about the benefits of high-performance DL models that can model temporal patterns in EHRs relative to existing CVD risk models. To fill the gap and test the generalizability of major statistical, ML, and DL models, we conducted comprehensive validation using procedures that are reflective of model performance under data shifts as well as internal validation with random train-test split.

We show that in the presence of large cohorts with comprehensive medical information, DL models outperform both conventional ML and linear statistical models in the prediction of HF, CHD, and stroke. This is to be expected; a sequential DL model maps the entire history of individuals to risk profiles whereas ML and statistical models rely on limited, expert-selected predictors and are oblivious to the temporal dimension of medical events. However, we encourage future work to replicate our work with potentially more advanced statistical and ML models. Our findings also corroborate similar studies that report no or minor differences between ML models and statistical models.^11,34^ Combined these findings underline that additional functional complexity, as seen in RF models compared to statistical models, has a minor effect on CVD risk predictions. Some recent studies claimed that the minimal performance gain in the ML models (i.e. RF) is due to the limited access to the EHR and those models can benefit from having more comprehensive features.^36^ However, even with the presence of the entire EHR, ML models still have to rely on the expert knowledge for predictor selection, which is still the main obstacle to further improve the performance. On the contrary, incorporating the temporal dimension of medical events and capturing the entire trajectory of patients, as seen in DL compared to ML and statistical, leads to major improvements.

The choice of model validation procedures distinguishes our study from others. Most of the available literature use randomly selected train-test sets for model validation,^31,37^ but this approach does not reflect the performance of models under data shifts, therefore it could lead to optimistic measures of performance. We evaluated the models under data shifts and showed that the performance of all models declined compared to validation with random train-test split. Although the DL model maintained the best performance, it had the largest performance decline compared to the statistical models when the test set was from a distinct region. This is because deep learning models can learn in-distribution associations very well, which however can lead to performance degradation when target population is mismatched (e.g. out-of-distribution datasets and datasets in the presence of data shifts).^38^ This should not be interpreted as overfitting; an overfitted model would not work well even for the in-distribution population, which is not the case in our experiment. Therefore, we conclude that data shifts can risk the generalizability of models, however, it does not necessarily link to overfitting. Similarly, the accuracy of all models declined as the time gap widened between the training cohort and validation cohort, especially for HF and stroke risk prediction. We showed that this can be remedied by regularly updating the model. Almost all models retained performance across different periods when updated every year. Lastly, our results suggest the calibration of models are generally sensitive to temporal changes. Models pertaining to HF risk prediction were subject to more severe calibration shift than the stroke and CHD. The DL models still achieved the best calibration; followed by the QRISK models, they substantially outperformed others. Our results also show that the calibration shift can potentially link to the prior probability shift: the bigger the prior probability shift, the larger the calibration shift. Model updating can only mitigate the concept shift but not the prior probability shift, therefore, its usefulness in alleviating the calibration shift is limited.

The present study also has several limitations. We focused on the risk of CHD, HF, and stroke. As such, the conclusions may not generalize to other diseases. Additionally, more hyperparameter searching for the models could have been considered, even though the reported models have achieved reasonable performance and lead to reasonable analyses. Another limitation is that censored patients were excluded from the analysis. Despite of the importance of survival framework in risk prediction, most of the DL models remained as binary classification model, thus, unable to handle the censored patients. To this end, some of the commonly used approaches are (i) discarding those patients and (ii) considering them as event free.^39^ The latter can substantially underestimate the risk in classification models but discarding censored patients will lead both survival and binary classification models to a similar risk prediction task and produce a more comparable result.^11^ Although this can yield a less representative cohort, the results are still valid in terms of model comparison. Our future work will explore the impact of survival framework vs. binary classification framework on risk prediction and the benefit of transforming DL binary classification models to DL survival models.

Improvement of CVD risk assessment in clinical care translates into better informed care and more timely interventions.^40,41^ With the growing availability of EHRs, this study supports the great potential of DL models for use in routine care and clinical management software to guide decision-making and regular system updating. For reproducibility, the code for all models and model evaluation strategies can be found in: github.com/deepmedicine/CVDRiskDistributionShift.

## Supplementary Material

ztac061_Supplementary_Data

## Data Availability

Scientific approval for this study was given by the Clinical Practice Research Datalink (CPRD) Independent Scientific Advisory Committee of UK (protocol number 16_049R). Data shared by consenting GP practices is de-identified and does not require individual patient consent for approved research (but individual patients can opt out from data sharing). The accessibility of the data is clearly explained on the website (https://www.cprd.com): ‘Access to data from Clinical Practice Research Datalink is subject to a full licence agreement containing detailed terms and conditions of use. Patient level datasets can be extracted for researchers against specific study specifications, following protocol approval from the Independent Scientific Advisory Committee (ISAC) of UK.’
