# Supplementary material for: Validation of risk prediction models applied to longitudinal electronic health record data for the prediction of major cardiovascular events in the presence of data shifts
Source: Eur Heart J Digit Health. 2022 Oct 21;3(4):535–47. doi: 10.1093/ehjdh/ztac061 (PMC9779795; doi:10.1093/ehjdh/ztac061)
Supplement: ztac061_Supplementary_Data [file ztac061_supplementary_data.zip › supplementary.docx]

# **Supplementary**

## **Contents**

Supplementary Methods

1. BEHRT
2. Details for BEHRT training
3. Details of predictor extraction and data imputation
4. Data imbalance

Supplementary Figures

Figure S1. BEHRT

Supplementary Tables

Table S1. Codes for the identification of LVH

Table S2. Parameters of Framingham, QRISK, ASSIGN-based random forest models

Table S3. Parameters for BEHRT

Table S4. Cohort baseline characteristics (out of region)

Table S5. Cohort baseline characteristics (temporal shift)

Table S6. Externally validated average precision on a cohort with patients from the non-random selected regions.

Table S7. Average precision of models under temporal data shifts.

## **Supplementary Methods**

### **BEHRT**

BEHRT (Figure S1) is a Transformer-based sequential model for EHR modelling(1). It includes four layers of features to represent a patient’s medical trajectory. They are event, age, segmentation, and positional code, respectively. The event layer includes the recorded medical events, and the rest three features together represent the chronological order of the events and the irregular interval between visits. More specifically, age represents the time stamp in the age that an event happens, segmentation uses two symbols (e.g., A and B) that change alternatively between two visits to assist the model distinguishing different visits, and the positional code is generated with a pre-defined function to indicate the chronological order of each event. Each layer is embedded with a separate embedding matrix, and the representation of each event is the summation of embeddings from all four layers of features. Afterwards, several layers of Transformer are followed for feature extraction. A pooling layer is used to pool the representation of the first-time step from the last layer of the Transformers for classification.

In our experiments, we included records from diagnoses, medications, lab tests, and procedures. Diagnoses used International classification of diseases, tenth revision (ICD-10(2)) level 4, and records from the primary care (Read(3)) were mapped to ICD-10 as described in (4). Medications were recorded in the British National Formulary coding scheme(5) at the section level. Lab tests were in Read code and only represented the type of lab test been carried. The procedure codes were recorded as the Office of Population Censuses and Surveys (OPCS) Classification of Interventions and Procedures codes. In total, there were 3858 diagnosis codes, 390 medication codes, 1439 lab test codes, and 679 procedure codes.

### **Details for BEHRT training**

BEHRT was implemented with Pytorch(6) and trained using Adam(7) optimizer with a three-stage learning rate scheduler(4) over 100 epochs on 2 GPUs using binary cross-entropy loss. The scheduler included 10%, 40%, and 50% for warm-up, hold, and cosine decay, respectively. We used batch size 32, hold learning rate 5e-5 with 4-step gradient accumulation. The early-stopping strategy was also included for training, and we stopped training once the validation loss did not decrease for 5 epochs. Additionally, for the out-of-time validation, BEHRT was trained on patients with baseline before 2000. However, because it was a substantially smaller cohort compared to the entire cohort, we initialised the model’s event embedding layer with the weighted trained for internal validation using the entire cohort, the rest of the model was randomly initialised.

### **Details of predictor extraction and data imputation**

The demographic factors, clinical diagnoses and medications, and clinical values were extracted from the GP and HES records. We identified all clinical diagnoses, except LVH, using a list of codes adapted from the CALIBER code repository(8). The predictors were defined the same way as described in the QRISK3.(9) The LVH was identified using codes representing the LVH verified by electrocardiogram test or diagnostic codes for LVH (Table S1). We identified all values recorded one year before the baseline for clinical values and calculated the mean for two or more recorded values.

In terms of data missingness, clinical diagnoses and medications were assumed to be present only if recorded. Smoking status and ethnicity were marked as unknown if not recoded. Missing data for body mass index (76%), systolic blood pressure (56%), total cholesterol to high density lipoprotein cholesterol ratio (85%), heart rate (96%) were imputed using multivariable imputation with chained equations(10).

### **Data Imbalance**

Because the outcomes investigated in this study were rare disease, therefore, the risk perdition tasks were potentially facing the data imbalance problem. Up-sampling and down-sampling are common strategies to handle such problem, however, in our study, there is not much difference in terms of the evaluation metrics when applying up-sampling for the risk prediction tasks or not. Additionally, applying up-sampling to the training can change the prior distribution and cause severe miscalibration, therefore, further model recalibration is required. Another study reported a similar finding as ours(11). Therefore, we concluded not to include any strategy to handle data imbalance in our study.

## **Supplementary Figures**


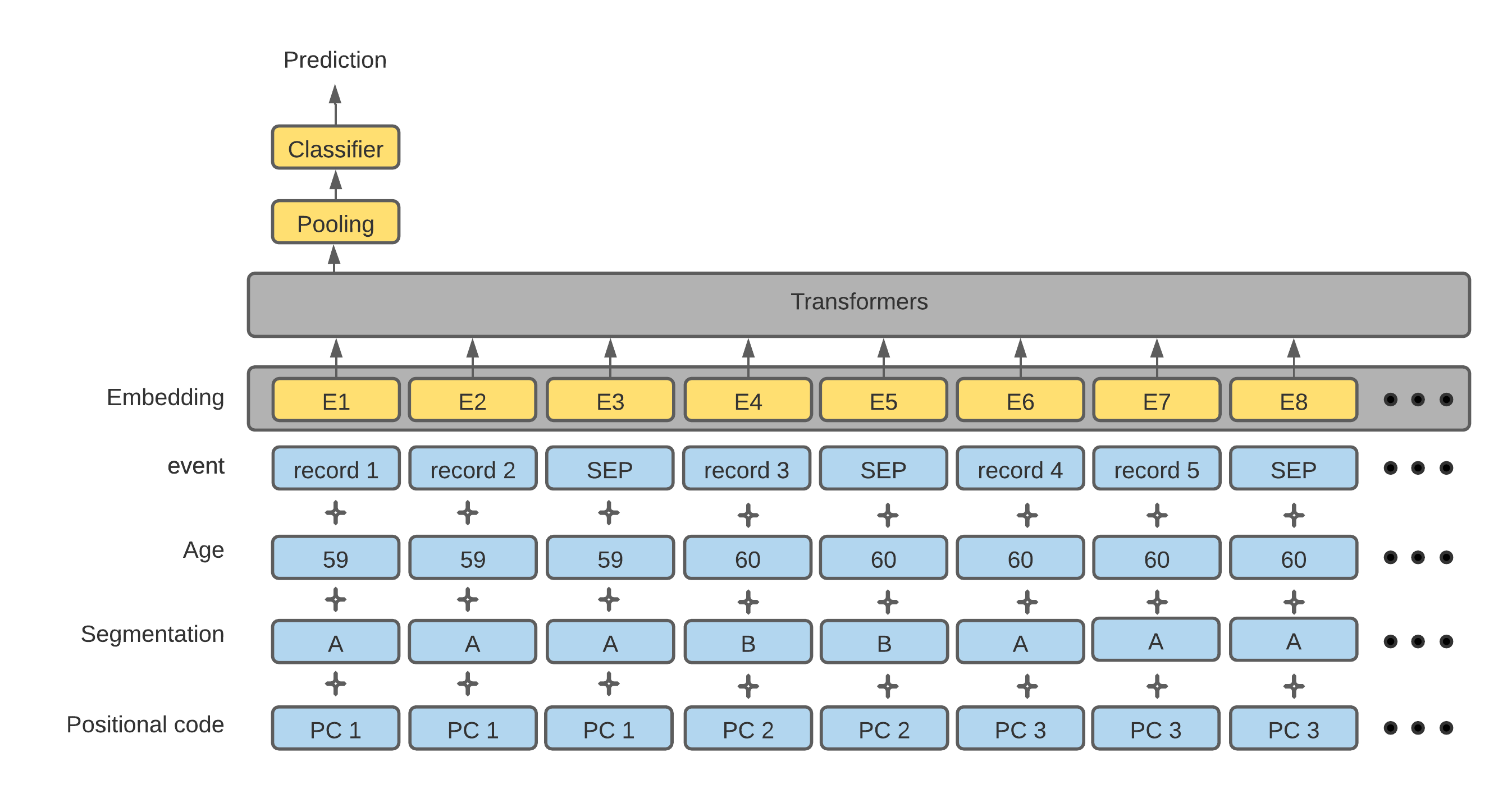


Figure S1. BEHRT model architecture. The representation of each event is the summation of embeddings from all four feature layers (event, age, segmentation, and positional code). The event includes recorded medical recorded, and symbol “SEP” is used to separate different visits. Age represents age in year for each event. Segmentation and positional code are auxiliary features to represent the order of different events.

## **Supplementary Tables**

**Table S1. Codes for the identification of LVH**

| Code | Type | Source | Description |
| --- | --- | --- | --- |
| 13857 | Medcode | CPRD (Test) | ECG: shows LVH |
| 23142 | Medcode | CPRD (Test) | ECG: LVH NOS |
| 6319 | Medcoe | CPRD (Test) | ECG: left ventricular hypertrophy |
| 526 | Medcode | CPRD (Clinical) | Left ventricular hypertrophy |

**Table S2. Parameters of Framingham, QRISK, ASSIGN-based random forest models**

| Parameters | Framingham | QRISK | ASSIGN |
| --- | --- | --- | --- |
| n_estimator | 800 | 800 | 800 |
| criterion | Gini | Gini | Gini |
| max_depth | 5 | 10 | 8 |
| max_features | auto | auto | auto |

**Table S3. Parameters for BEHRT**

| Max sequence length | 512 | Hidden size | 256 |
| --- | --- | --- | --- |
| No. of layers | 6 | Hidden activation | gelu |
| Intermediate size | 512 | No. of attention heads | 8 |
| Dropout probability | 0.2 |  |  |

**Table S4. Cohort baseline characteristics (out of region)**

| Predictor | | Training | Validation |
| --- | --- | --- | --- |
| No. of patients | | 838,961 | 257,314 |
| % of HF patients | | 3.3 | 3.6 |
| % of stroke patients | | 6.8 | 6.8 |
| % of CHD patiennts | | 10.2 | 10.9 |
| Sex, n (%) | |  |  |
|  | Male | 409,522 (48.8) | 125,105 (48.6) |
|  | Female | 429,409 (51.2) | 132,209 (51.4) |
| Age, mean (SD) | | 58.2 (15.4) | 57.3 (15.0) |
| IMD score, mean (SD) | | 2.6 (1.3) | 3.1 (1.4) |
| Family history of CHD, n (%) | | 246,416 (29.4) | 94,626 (36.7) |
| BMI, mean (SD) | | 27.6 (3.2) | 27.7 (3.2) |
| Strategic health authority (region), n (%) | |  |  |
|  | North East | - | 27,195 (2.5) |
|  | North West | - | 175,416 (16.0) |
|  | Yorkshire and the Humber | - | 54,655 (5.0) |
|  | East Midlands | 38,242 (3.5) | - |
|  | West Midlands | 137,824 (12.6) | - |
|  | East of England | 134,314 (12.3) | - |
|  | South West | 130,170 (11.9) | - |
|  | South Central | 136,189 (12.4) | - |
|  | London | 126,175 (11.5) | - |
|  | South East Coast | 136,047 (12.4) | - |
| Ethnicity, n (%) | |  |  |
|  | Unknown | 536,157 (63.9) | 147,804 (57.4) |
|  | White | 291,908 (34.8) | 107,625 (41.8) |
|  | Other Asian | 905 (0.1) | 111 (0.0) |
|  | Pakistani | 802 (0.1) | 435 (0.2) |
|  | Indian | 2,794 (0.3) | 276 (0.1) |
|  | Other | 2,603 (0.3) | 559 (0.2) |
|  | Caribbean | 1,562 (0.2) | 97 (0.0) |
|  | Mixed | 697 (0.1) | 93 (0.0) |
|  | Bangladeshi | 283 (0.0) | 55 (0.0) |
|  | Chinese | 495 (0.1) | 161 (0.1) |
|  | Black African | 755 (0.1) | 98 (0.0) |
| Smoking status, n (%) | |  |  |
|  | Not recorded | 596,842 (71.1) | 180,085 (70.0) |
|  | Non-smoker | 119,100 (14.2) | 35,891 (13.9) |
|  | Ex-smoker | 81,872 (9.8) | 24,640 (9.6) |
|  | Light smoker (<10 cigarettes/day) | 11,436 (1.4) | 4,140 (1.6) |
|  | Moderate smoker (10-20 cigarettes/ day) | 16,619 (2.0) | 6,650 (2.6) |
|  | Heavy smoker (>20 cigarettes/day) | 13,092 (1.6) | 5,908 (2.3) |
| Clinical values | |  |  |
|  | Systolic blood pressure, mean (SD) | 135.4 (13.1) | 135.3 (12.9) |
|  | Cholesterol/HDL | 3.98 (0.69) | 4.00 (0.63) |
| Comorbidity, n (%) | |  |  |
|  | Diabetes | 29,478 (3.5) | 9,494 (3.7) |
|  | Rheumatoid arthritis | 5,584 (0.7) | 1,923 (0.7) |
|  | Atrial fibrillation | 26,804 (3.2) | 7,333 (2.8) |
|  | Chronic kidney disease | 5,222 (0.6) | 1,505 (0.6) |
|  | Migraine | 25,872 (3.1) | 8,917 (3.5) |
|  | Severe mental illness | 4,460 (0.5) | 1,653 (0.6) |
|  | Systemic lupus erythematosus | 667 (0.1) | 280 (0.1) |
|  | HIV or AIDS | 1,316 (0.2) | 439 (0.2) |
|  | Erectile dysfunction | 21,421 (2.6) | 6,464 (2.5) |
| Prescribed medication, n (%) | |  |  |
|  | Treated hypertension | 177,009 (21.1) | 56,047 (21.8) |
|  | Antipsychotic | 4,381 (0.5) | 1,397 (0.5) |
|  | Corticosteroid | 32,983 (3.9) | 11,405 (4.4) |
| HDL, high-density lipoprotein; IMD, Index of Multiple Deprivation; SD, standard deviation | | | |

**Table S5. Cohort baseline characteristics (temporal shift)**

| Predictor | | Baseline (year) | | | | | | | | | |
| --- | --- | --- | --- | --- | --- | --- | --- | --- | --- | --- | --- |
|  |  | 1999 | 2000 | 2001 | 2002 | 2003 | 2004 | 2005 | 2006 | 2007 | 2008 |
| No. of patients | | 39,479 | 45,438 | 60,288 | 77,133 | 83,845 | 93,496 | 94,228 | 92,344 | 86,853 | 78,444 |
| % of HF patients | | 4.7 | 4.4 | 4.0 | 3.3 | 2.7 | 2.3 | 2.1 | 1.9 | 1.8 | 2.0 |
| % of stroke patients | | 7.9 | 7.7 | 7.5 | 7.1 | 6.2 | 5.7 | 5.1 | 4.8 | 4.7 | 4.8 |
| % of CHD patients | | 10.6 | 11.2 | 11.8 | 11.1 | 10.0 | 9.2 | 8.5 | 8.2 | 7.8 | 8.3 |
| Sex, n (%) | |  |  |  |  |  |  |  |  |  |  |
|  | Male | 19,181 (48.6) | 22,030 (48.5) | 29,212 (48.5) | 37,832 (49.0) | 41,026 (48.9) | 45,538 (48.7) | 45,910 (48.7) | 44,835 (48.6) | 42,360 (48.8) | 38,340(48.9) |
|  | Female | 20,298 (51.4) | 23,408 (51.5) | 31,076 (51.5) | 39,301 (51.0) | 42,819 (51.1) | 47,958 (51.3) | 48,318 (51.3) | 47,509 (51.4) | 44,493 (51.2) | 40,104 (51.1) |
| Age, mean (SD) | | 58.8 (15.8) | 58.9 (15.5) | 58.9 (15.6) | 58.5 (15.5) | 58.0 (15.4) | 57.7 (15.2) | 57.2 (15.0) | 56.7 (14.9) | 56.7 (14.9) | 56.6 (15.0) |
| IMD score, mean (SD) | | 2.8 (1.4) | 2.8 (1.4) | 2.7 (1.4) | 2.7 (1.4) | 2.7 (1.4) | 2.7 (1.4) | 2.7 (1.4) | 2.7 (1.4) | 2.7 (1.4) | 2.7 (1.4) |
| Family history of CHD, n (%) | | 15,548 (39.4) | 17,397 (38.3) | 21,327 (35.4) | 24,961 (32.4) | 26,169 (31.2) | 27,849 (29.8) | 26,750 (28.4) | 25,170 (27.3) | 23,135 (26.6) | 20,542 (26.2) |
| BMI, mean (SD) | | 27.5 (2.7) | 27.5 (2.6) | 27.5 (2.6) | 27.6 (2.8) | 27.6 (2.9) | 27.6 (3.1) | 27.6 (3.5) | 27.7 (3.6) | 27.8 (3.6) | 27.8 (3.6) |
| Smoking status, n (%) | |  |  |  |  |  |  |  |  |  |  |
|  | Not recorded | 35,864 (90.8) | 40,444 (89.0) | 51,898 (86.1) | 64,708 (83.4) | 65,356 (77.9) | 59,718 (63.9) | 59,287 (62.9) | 52,291 (56.6) | 51,435 (59.2) | 44,061 (56.2) |
|  | Non-smoker | 1,853 (4.7) | 2,452 (5.4) | 4,012 (6.7) | 5,922 (7.7) | 9,214 (10.9) | 17,223 (18.4) | 17,395 (18.5) | 18,740 (20.3) | 16,270 (18.7) | 16,004 (20.4) |
|  | Ex-smoker | 877 (2.2) | 1,362 (3.0) | 2,487 (4.1) | 3,916 (5.1) | 5,600 (6.7) | 10,553 (11.3) | 11,417 (12.1) | 14,585 (15.8) | 13,207 (15.2) | 12,940 (16.4) |
|  | Light smoker (<10 cigarettes/day) | 233 (0.6) | 299 (0.7) | 491 (0.8) | 638 (0.8) | 920 (1.1) | 1,537 (1.6) | 1,608 (1.7) | 1,825 (2.0) | 1,690 (1.9) | 1,567 (2.0) |
|  | Moderate smoker (10-20 cigarettes/ day) | 346 (0.9) | 488 (1.1) | 724 (1.2) | 990 (1.3) | 1,469 (1.8) | 2,422 (2.6) | 2,403 (2.6) | 2,747 (3.0) | 2,434 (2.8) | 2,205 (2.8) |
|  | Heavy smoker (>20 cigarettes/day) | 306 (0.8) | 393 (0.9) | 676 (1.1) | 959 (1.2) | 1,286 (1.5) | 2,043 (2.2) | 2,118 (2.2) | 2,156 (2.3) | 1,817 (2.1) | 1,667 (2.1) |
| Clinical values | |  |  |  |  |  |  |  |  |  |  |
|  | Systolic blood pressure, mean (SD) | 137.4 (13.3) | 137.3 (13.4) | 136.9 (13.4) | 136.6 (13.4) | 136.2 (13.5) | 135.6 (13.4) | 134.8 (13.0) | 133.8 (12.5) | 133.5 (12.3) | 133.1 (12.1) |
|  | Cholesterol/HDL | 3.99 (0.51) | 3.99 (0.49) | 3.99 (0.54) | 4.00 (0.57) | 4.00 (0.62) | 4.00 (0.68) | 3.99 (0.73) | 3.98 (0.78) | 4.0 (0.81) | 3.99 (0.79) |
| Comorbidity, n (%) | |  |  |  |  |  |  |  |  |  |  |
|  | Diabetes | 484 (1.2) | 654 (1.4) | 1,048 (1.7) | 1,626 (2.1) | 2,271 (2.7) | 3,138 (3.4) | 3,578 (3.8) | 4,629 (5.0) | 4,758 (5.5) | 4,518 (5.8) |
|  | Rheumatoid arthritis | 265 (0.7) | 278 (0.6) | 361 (0.6) | 454 (0.6) | 514 (0.6) | 620 (0.7) | 678 (0.7) | 640 (0.7) | 618 (0.7) | 579 (0.7) |
|  | Atrial fibrillation | 883 (2.2) | 1,242 (2.7) | 1,668 (2.8) | 2,202 (2.9) | 2,441 (2.9) | 2,805 (3.0) | 2,855 (3.0) | 2,939 (3.2) | 2,837 (3.3) | 2,735 (3.5) |
|  | Chronic kidney disease | 83 (0.2) | 138 (0.3) | 204 (0.3) | 301 (0.4) | 411 (0.5) | 470 (0.5) | 491 (0.5) | 679 (0.7) | 700 (0.8) | 686 (0.9) |
|  | Migraine | 1,188 (3.0) | 1,324 (2.9) | 1,669 (2.8) | 2,030 (2.6) | 2,289 (2.7) | 2,679 (2.9) | 2,938 (3.1) | 3,334 (3.6) | 3,258 (3.8) | 3,195 (4.1) |
|  | Severe mental illness | 181 (0.5) | 232 (0.5) | 308 (0.5) | 373 (0.5) | 396 (0.5) | 453 (0.5) | 599 (0.6) | 558 (0.6) | 484 (0.6) | 529 (0.7) |
|  | Systemic lupus erythematosus | 34 (0.1) | 31 (0.1) | 50 (0.1) | 58 (0.1) | 60 (0.1) | 83 (0.1) | 88 (0.1) | 92 (0.1) | 89 (0.1) | 76  (0.1) |
|  | HIV or AIDS | 64 (0.2) | 74 (0.2) | 76 (0.1) | 105 (0.1) | 138 (0.2) | 136 (0.1) | 131 (0.1) | 1511 (0.2) | 176 (0.2) | 119 (0.2) |
|  | Erectile dysfunction | 450 (1.1) | 631 (1.4) | 942 (1.5) | 1,357 (1.8) | 1,830 (2.1) | 2,309 (2.5) | 2,614 (2.7) | 3,103 (3.4) | 3,204 (3.7) | 3,122 (4.0) |
| Prescribed medication, n (%) | |  |  |  |  |  |  |  |  |  |  |
|  | Treated hypertension | 6,933 (17.6) | 8,558 (18.8) | 11,617 (19.3) | 15,176 (19.7) | 17,445 (20.8) | 20,781 (22.2) | 21,916 (23.3) | 22,506 (24.4) | 21,314 (24.5) | 18,850 (24.0) |
|  | Antipsychotic | 58 (0.1) | 88 (0.2) | 175 (0.3) | 260 (0.3) | 418 (0.5) | 490 (0.5) | 629 (0.7) | 741 (0.8) | 766 (0.9) | 762 (1.0) |
|  | Corticosteroid | 1,499 (3.8) | 1,745 (3.8) | 2,151 (3.6) | 2,699 (3.5) | 3,137 (3.7) | 3,630 (3.9) | 4,014 (4.3) | 4,447 (4.8) | 4,153 (4.8) | 3,776 (4.8) |
| HDL, high-density lipoprotein; IMD, Index of Multiple Deprivation; SD, standard deviation | | | | | | | | | | | |

**Table S6. Externally validated average precision on a cohort with patients from the non-random selected regions.**

|  | Average precision | | |
| --- | --- | --- | --- |
| Models | HF | Stroke | CHD |
| BEHRT | 0.501 | 0.740 | 0.760 |
| QRISK3 | 0.254 | 0.408 | 0.453 |
| RF (QRISK) | 0.266 | 0.439 | 0.477 |
| ASSIGN | 0.215 | 0.341 | 0.382 |
| RF (ASSIGN) | 0.225 | 0.347 | 0.395 |
| Framingham | 0.254 | 0.427 | 0.421 |
| RF (Framingham) | 0.246 | 0.421 | 0.431 |

**Table S7. Average precision of models under temporal data shifts.**

|  | Baseline (year) | | | | | | | | | |
| --- | --- | --- | --- | --- | --- | --- | --- | --- | --- | --- |
|  | 1999 | 2000 | 2001 | 2002 | 2003 | 2004 | 2005 | 2006 | 2007 | 2008 |
| Model | Heart failure | | | | | | | | | |
| BEHRT | 0.639 | 0.519 | 0.480 | 0.467 | 0.410 | 0.363 | 0.338 | 0.300 | 0.282 | 0.305 |
| QRISK3 | 0.355 | 0.332 | 0.303 | 0.294 | 0.257 | 0.207 | 0.218 | 0.178 | 0.181 | 0.183 |
| RF (QRISK) | 0.475 | 0.324 | 0.304 | 0.289 | 0.246 | 0.211 | 0.218 | 0.172 | 0.180 | 0.193 |
| ASSIGN | 0.286 | 0.275 | 0.261 | 0.238 | 0.203 | 0.174 | 0.174 | 0.138 | 0.139 | 0.148 |
| RF (ASSIGN) | 0.413 | 0.273 | 0.264 | 0.238 | 0.200 | 0.177 | 0.177 | 0.140 | 0.135 | 0.149 |
| Framingham | 0.327 | 0.298 | 0.83 | 0.266 | 0.229 | 0.192 | 0.199 | 0.154 | 0.162 | 0.170 |
| RF (Framingham) | 0.328 | 0.291 | 0.270 | 0.253 | 0.217 | 0.180 | 0.178 | 0.140 | 0.147 | 0.153 |
|  | Stroke | | | | | | | | | |
| BEHRT | 0.773 | 0.723 | 0.738 | 0.730 | 0.714 | 0.692 | 0.663 | 0.660 | 0.648 | 0.658 |
| QRISK3 | 0.413 | 0.434 | 0.370 | 0.456 | 0.430 | 0.412 | 0.383 | 0.381 | 0.378 | 0.376 |
| RF (QRISK) | 0.520 | 0.466 | 0.506 | 0.483 | 0.457 | 0.440 | 0.407 | 0.397 | 0.387 | 0.389 |
| ASSIGN | 0.377 | 0.407 | 0.425 | 0.404 | 0.370 | 0.345 | 0.303 | 0.295 | 0.285 | 0.276 |
| RF (ASSIGN) | 0.475 | 0.403 | 0.415 | 0.394 | 0.368 | 0.343 | 0.302 | 0.292 | 0.289 | 0.272 |
| Framingham | 0.419 | 0.457 | 0.486 | 0.470 | 0.445 | 0.427 | 0.390 | 0.383 | 0.381 | 0.368 |
| RF (Framingham) | 0.433 | 0.448 | 0.463 | 0.485 | 0.441 | 0.426 | 0.397 | 0.385 | 0.384 | 0.375 |
|  | Coronary heart disease | | | | | | | | | |
| BEHRT | 0.764 | 0.690 | 0.726 | 0.719 | 0.711 | 0.696 | 0.680 | 0.661 | 0.652 | 0.664 |
| QRISK3 | 0.388 | 0.424 | 0.452 | 0.444 | 0.420 | 0.399 | 0.376 | 0.360 | 0.348 | 0.373 |
| RF (QRISK) | 0.505 | 0.449 | 0.492 | 0.485 | 0.468 | 0.447 | 0.425 | 0.407 | 0.390 | 0.411 |
| ASSIGN | 0.354 | 0.384 | 0.415 | 0.405 | 0.381 | 0.366 | 0.341 | 0.330 | 0.319 | 0.343 |
| RF (ASSIGN) | 0.470 | 0.393 | 0.421 | 0.411 | 0.382 | 0.367 | 0.348 | 0.332 | 0.321 | 0.335 |
| Framingham | 0.361 | 0.390 | 0.430 | 0.423 | 0.396 | 0.384 | 0.362 | 0.354 | 0.346 | 0.369 |
| RF (Framingham) | 0.379 | 0.405 | 0.453 | 0.440 | 0.419 | 0.398 | 0.380 | 0.363 | 0.354 | 0.370 |

# References

1. Li Y, Rao S, Solares JRA, Hassaine A, Ramakrishnan R, Canoy D, et al. BEHRT: Transformer for Electronic Health Records. Sci Rep [Internet]. 2020;10(1):7155. Available from: https://doi.org/10.1038/s41598-020-62922-y

2. Organization WH. ICD-10 : international statistical classification of diseases and related health problems : tenth revision. 2nd ed. World Health Organization; 2004. p. Spanish version, 1st edition published by PAHO as.

3. Chisholm J. The Read clinical classification. BMJ Br Med J. 1990;300(6732):1092.

4. Li Y, Mamouei M, Salimi-khorshidi G, Rao S, Hassaine A, Canoy D, et al. Hi-BEHRT : Hierarchical Transformer-based model for accurate prediction of clinical events using multimodal longitudinal electronic health records. :1–11.

5. Joint Formulary Committee. British National Formulary. BMJ Group and Pharmaceutical Press.

6. Paszke A, Gross S, Massa F, Lerer A, Bradbury J, Chanan G, et al. Pytorch: An imperative style, high-performance deep learning library. In: Advances in neural information processing systems. 2019. p. 8026–37.

7. Kingma DP, Ba J. Adam: A method for stochastic optimization. arXiv Prepr arXiv14126980. 2014;

8. Kuan V, Denaxas S, Gonzalez-Izquierdo A, Direk K, Bhatti O, Husain S, et al. A chronological map of 308 physical and mental health conditions from 4 million individuals in the English National Health Service. Lancet Digit Heal. 2019;1(2):e63–77.

9. Hippisley-Cox J, Coupland C, Brindle P. Development and validation of QRISK3 risk prediction algorithms to estimate future risk of cardiovascular disease: prospective cohort study. BMJ. 2017 May;357:j2099.

10. Van Buuren S, Groothuis-Oudshoorn K. mice: Multivariate imputation by chained equations in R. J Stat Softw. 2011;45(1):1–67.

11. van den Goorbergh R, van Smeden M, Timmerman D, Van Calster B. The harm of class imbalance corrections for risk prediction models: illustration and simulation using logistic regression. J Am Med Informatics Assoc [Internet]. 2022 Jun 10;ocac093. Available from: https://doi.org/10.1093/jamia/ocac093
