# Supplementary material for: Validation of risk prediction models applied to longitudinal electronic health record data for the prediction of major cardiovascular events in the presence of data shifts
Source: Eur Heart J Digit Health. 2022 Oct 21;3(4):535–47. doi: 10.1093/ehjdh/ztac061 (PMC9779795; doi:10.1093/ehjdh/ztac061)
Supplement: ztac061_Supplementary_Data [file ztac061_supplementary_data.zip › Translational Perspective.docx]

Cardiovascular disease (CVD) risk models have a long tradition in clinical care. Despite rapid advances in modelling over the past decade and the increasing availability of longitudinal electronic health records, CVD risk models proposed in 1990s and 2000s are still routinely used across the world with minor changes. In line with the TRIPOD statement and using representative data from 3.05 million individuals in the UK, we performed a rigorous comparison of novel deep learning and machine learning models with conventional CVD risk models. The findings highlight the merits and shortcomings of these models in terms of predictive performance.
