# Supplementary material for: Validation of risk prediction models applied to longitudinal electronic health record data for the prediction of major cardiovascular events in the presence of data shifts
Source: Eur Heart J Digit Health. 2022 Oct 21;3(4):535–47. doi: 10.1093/ehjdh/ztac061 (PMC9779795; doi:10.1093/ehjdh/ztac061)
Supplement: ztac061_Supplementary_Data [file ztac061_supplementary_data.zip › Permissions.docx]

The authors do hereby declare that all illustrations and figures in the manuscript are original and not require reprint permission.
